# Re-conceptualising and accounting for examiner (cut-score) stringency in a ‘high frequency, small cohort’ performance test

**DOI:** 10.1007/s10459-020-09990-x

**Published:** 2020-09-02

**Authors:** Matt Homer

**Affiliations:** Leeds Institute of Medical Education, School of Medicine, University of Leeds, Leeds, LS2 9JT UK

**Keywords:** Borderline regression method, Cut-scores, Examiner stringency, Hawks and doves, OSCE

## Abstract

Variation in examiner stringency is an ongoing problem in many performance settings such as in OSCEs, and usually is conceptualised and measured based on scores/grades examiners award. Under borderline regression, the standard within a station is set using checklist/domain scores and global grades acting in combination. This complexity requires a more nuanced view of what stringency might mean when considering sources of variation of cut-scores in stations. This study uses data from 349 administrations of an 18-station, 36 candidate single circuit OSCE for international medical graduates wanting to practice in the UK (PLAB2). The station-level data was gathered over a 34-month period up to July 2019. Linear mixed models are used to estimate and then separate out examiner (n = 547), station (n = 330) and examination (n = 349) effects on borderline regression cut-scores. Examiners are the largest source of variation in cut-scores accounting for 56% of variance in cut-scores, compared to 6% for stations, < 1% for exam and 37% residual. Aggregating to the exam level tends to ameliorate this effect. For 96% of examinations, a ‘fair’ cut-score, equalising out variation in examiner stringency that candidates experience, is within one standard error of measurement (SEM) of the actual cut-score. The addition of the SEM to produce the final pass mark generally ensures the public is protected from almost all false positives in the examination caused by examiner cut-score stringency acting in candidates’ favour.

## Introduction

### Examiner stringency as an effect on scores

There is a longstanding interest on the effect of examiners on standards in performance assessments like OSCEs (Bartman et al. 2013; Downing 2005; Fuller et al. 2017; Harasym et al. 2008; Jefferies et al. 2007; McManus et al. 2006; Pell et al. 2010; Yeates et al. 2018; Yeates and Sebok-Syer 2017). Variation in examiner stringency is usually conceptualised and measured based on the scores (or grades) that examiners produce within stations—i.e. examiners might be considered ‘hawks’ if their scores are systematically lower than those of other examiners. However, in many assessment designs examiners are clustered in circuits, and ‘see’ different groups of candidates, which make it difficult to disentangle examiner, station and candidate effects on scores/grades.

Attempts to investigate examiner score stringency have usually rested on having sufficient ‘linking’ of patterns of scoring, either through having a sufficiently large data set of examiner scores for a bank of stations (McManus et al. 2006), or via experimental design—for example, having sufficient numbers of examiners watch videos of performance (Yeates et al. 2018) to provide connections between scoring across different parallel circuits in an exam. In either case, examiner score stringency can be estimated using statistical modelling approaches to disentangle the effects of examiners, or cohorts of examiners (Yeates and Sebok-Syer 2017), and stations. This work generally finds that variation in examiner stringency is sufficiently important to have a discernible effect on examination outcomes—of the order of 5% of candidates might have had different pass/fails decisions on removal of the effect of differential examiner stringency (McManus et al. 2006; Yeates et al. 2018).

### Re-conceptualising examiner stringency as an effect on cut-scores

**Fig. 1:**
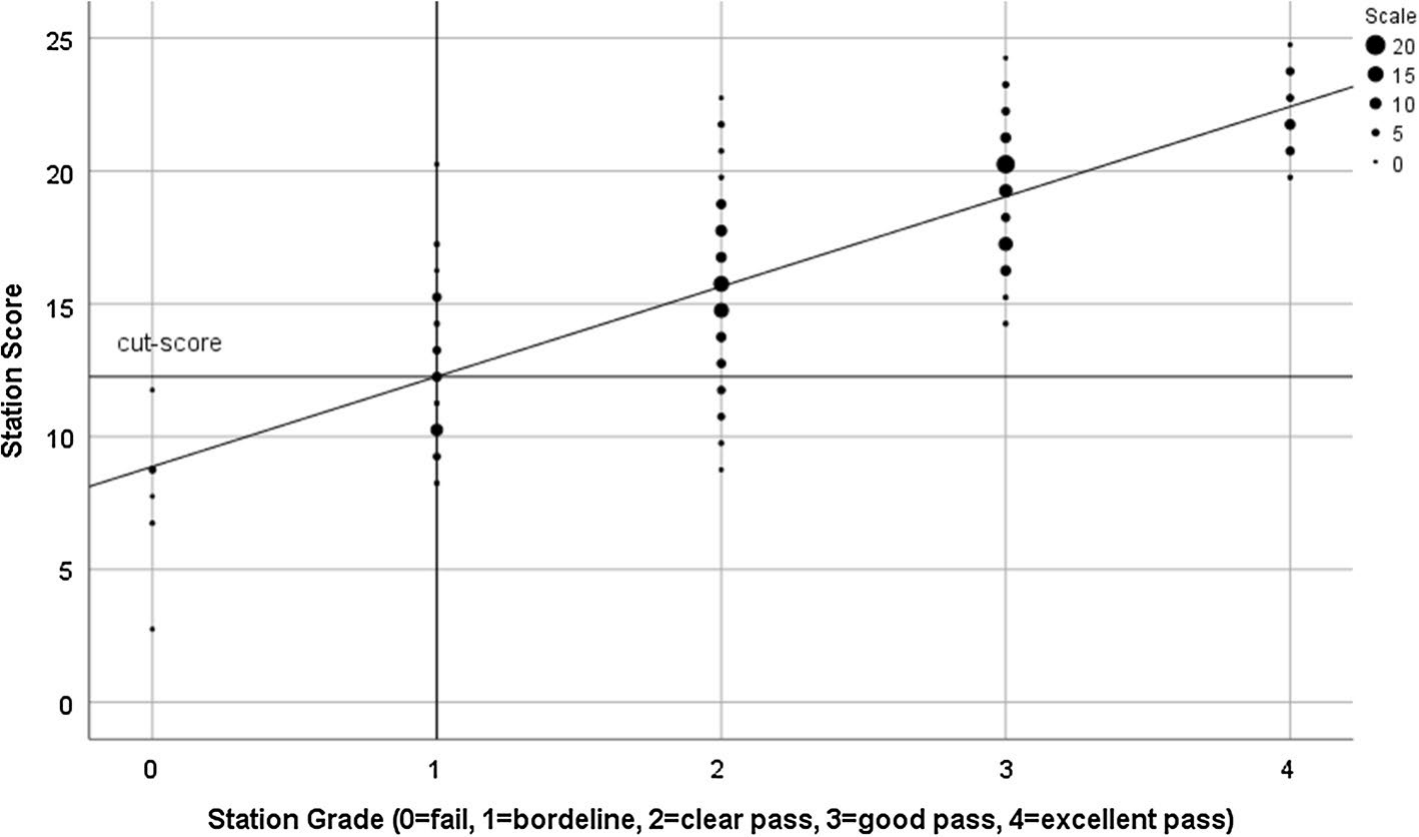
Scores (y) regressed on grades (x) to derive station cut-score under BRM

Under examinee-centred methods of standard setting, such as the widely used borderline regression method (BRM) (Kramer et al. 2003; McKinley and Norcini 2014; Pell et al. 2010), the cut-score in a station is calculated through the combined effect of scores/grades via the regression modelling of grades on scores, rather than being based on either of these alone. Figure 1 shows a scatter plot for a hypothetical station with scores on the vertical (y) axis, and grades horizontal (x-axis). The station cut-score under BRM is the station score predicted by the simple regression line for the borderline grade (x = 1).

One could imagine that some examiners might produce patterns of scores and grades that lead to systematically different (i.e. higher or lower) BRM cut-scores compared to those of other examiners—whether assessing the same or different stations. For example, and for whatever reason, a hypothetical examiner might be hawkish on checklist scores, whilst neutral on grades. This would impact on the BRM cut-score—in this case, tending to lower it via the regression modelling (see Fig. 1). There are alternative hypothetical patterns of examiner behaviour—where differential levels of stringency in scores/grades impact differently on BRM cut-scores.

Regardless of the precise mechanism in play for a particular examiner, this *cut*-*score stringency* could be different to how the same examiners might be ranked in terms of stringency based solely on the scores or grades they award (*score stringency*). For clarity, we define these key terms explicitly as follows:

**Table:** 

*Examiner score stringency*	Variation in station scores (or grades) across examiners (often termed as ‘hawks and doves’)
*Examiner cut*-*score score stringency*	Variation in station cut-scores across examiners under an examinee-centred scoring method such as BRM

Quantification of the extent to which examiner cut-score stringency, rather than examiner stringency in scores/grades, exists via BRM standard setting is an under-researched area. To the author’s best knowledge, there is no literature on this issue, but it is one that is directly relevant to the key purpose of assessments—deciding dependably and fairly between passing and failing candidates (Cizek and Bunch 2007, Chapter 1). As the proceeding discussion suggests, the interplay between patterns of scoring across groups of examiners and stations, and the resultant sets of BRM cut-scores, is clearly a complex phenomenon, and one that can only be explored with data consisting of many observations of examiners in many stations across many examination sittings, or via complex and potentially resource intensive intervention studies.

### This purposes of this paper

This paper begins to investigate examiner cut-score stringency, using statistical modelling to separate out and estimate examiner, station and examination effects on cut-scores derived using BRM. The over-arching research questions of this study are:

- What is the extent of variation in station-level examiner cut-score stringency, in comparison to that of stations and examinations?
- What impact on exam-level cut-scores does station-level examiner cut-score stringency have?

The study uses station-level cut-score data from 6214 station administrations from the PLAB2 examination in the UK (General Medical Council 2020a), a small-cohort OSCE (hence the ‘high frequency, small cohort’ phraseology of the title). In essence, to answer the research questions, we estimate examiner and other effects (station and examination) on cut-scores across this large set of stations, and then use the model to create ‘fair’ cut-scores at the exam level. We use a mixed effects modelling methodology to do this (Bates et al. 2015). More details of the research context follow in the first sub-section of the methodology that follows.

## Methodology

### Research context: PLAB2

The Professional and Linguistic Assessments Board test, or the PLAB test, is designed to ensure that doctors who have qualified abroad have the appropriate set of knowledge and skills to begin practising medicine in the UK (General Medical Council 2020b) at the level equivalent to that at the end of the first year of Foundation Training (i.e. first year of clinical practice). There are two parts to the PLAB test, an applied knowledge test (PLAB1) and an 18 station OSCE (PLAB2).

PLAB2 consists of 18 stations designed to reflect real life clinical settings such as patient consultation in primary care, or practice on an acute NHS ward. It measures the extent to which candidates can apply their knowledge to provide good care to patients (General Medical Council 2020a). Clinically trained examiners are randomly assigned to stations, and score candidates via a holistic judgement of the performance in a four-point global grade (0 = unsatisfactory, 1 = borderline, 2 = satisfactory, 3 = good). Examiners also score candidates 1 to 4 in each station in three separate domains (Data gathering, technical and assessment skills, Clinical management skills, and Interpersonal skills) to give a total station domain score out of 12.

Each PLAB2 administration in this study consists of a morning and afternoon circuit with the same examiner in each station, and all assessment outcomes for the maximum of 36 candidates are combined for the BRM standard setting for the day. The large volume of PLAB2 tests administered (for example, 169 in the calendar year 2019) require an examinee-centred standard setting approach, and BRM is used to regress total station scores on global grades to set the cut-score in the station in a way similar to that shown in Fig. 1. Examination-level cut-scores are produced by aggregating across the 18 stations, and a standard error of measurement (SEM) (Hays et al. 2008) is then added to this to produce the final pass-mark—this has a dual purpose of limiting both compensation across stations and the number of false positive decisions.

Details on the sample of examiners, stations and administrations is given in the next sub-section, followed by the statistical methods used in this study.

### Data sample

The PLAB2 data employed in this study is at station level, and in total there are 6214 station administrations from 349 examinations administered over the period February 2016 to July 2019.

For illustrative purposes, Table 1 shows a simulated subset of sample data. For Exam 1 there are 18 rows, one per station in that exam. Bolded cells exemplify how facets (stations and examiners) might repeat over the dataset. The key interest in this paper is estimating how these facets impact on station cut-scores.

**Table 1:** PLAB2 data snippet (simulated)

Row	Examination	Examiner ID	Station ID	Cut-score
1	Exam1	9443	112	5.85
2	Exam1	9684	279	5.94
3	Exam1	6186	300	4.94
4	Exam1	**5438**	125	4.09
5	Exam1	9234	393	6.96
6	Exam1	4105	357	4.07
7	Exam1	4583	24	6.31
8	Exam1	8710	5	7.05
9	Exam1	6515	349	4.50
10	Exam1	5813	328	6.45
11	Exam1	8343	326	4.81
12	Exam1	9523	112	6.04
13	Exam1	2910	**386**	4.81
14	Exam1	1589	448	7.77
15	Exam1	5184	162	4.68
16	Exam1	2214	397	7.12
17	Exam1	3748	414	5.65
18	Exam1	428	2	6.88
19	Exam2	5978	**386**	4.78
20	Exam2	**5438**	284	5.63
…	…	…	…	…
…	…	…	…	…

The station-level cut-scores (on a 12 point scale) are close to a normal distribution, and a have mean of 5.56 = 46.3%, and median 5.50 = 45.8%.

Separate analysis not included here indicates that the typical internal consistency reliability of the examination is on average relatively high (mean alpha = 0.76 for station scores across the 349 exams), and BRM has been shown to work well in this setting (Homer et al. 2019). Candidate level data was not available for analysis, an issue we will return to at relevant points in the paper.

Table 2 summarises the frequency of each facet considered in this analysis (examiner, station, examination).

**Table 2:** Sample sizes and descriptives for the three facets in the PLAB2 data

	N	Mean	Minimum	Maximum	Percentiles		
					25	50	75
Examiner	547	11.36	1	123	3	6	13
Station	330	18.83	1	68	8	17	27
Examination	349	17.81	16	18	18	18	18

We see, for example, that across the 6214 stations administrations, there were 547 different examiners and 330 different stations. It is also clear from Table 2 that both individual examiners, and stations, are present in the data in varying degrees, but that typically there are multiple data points for each level of each facet—median 6 and 17 for examiner and station respectively. This gives us some confidence that there is sufficient data to estimate effects on cut-scores with some degree of precision.

Table 2 also shows that on occasion individual stations were supressed from the intended 18 station OSCE—the mean no. of stations per exam is 17.81 (i.e. less than 18). These stations were removed from the examination—usually, because of problems observed during the examination which meant the pattern of scores/grades were deemed insufficiently reliable for use in this high-stakes setting (Pell et al. 2010).

The exact nature of the calculation of the station-level cut-score using BRM has been modified in PLAB2 over the course of the period 2016–2019. In more recent years, the x-value used to create the pass mark has been increased a little above the usual ‘borderline’ value of 1 (see Fig. 1) increasing cut-scores. However, to keep all the data directly comparable we have consistently used the original approach to BRM in all that follows. Actual cut-scores in PLAB2 are typically higher than those shown in this work. This issue does not effect in any way the substantive findings presented.

This study does not directly employ candidate scores—these were not available for analysis. In extant work on examiner stringency (McManus et al. 2006) candidate variation is often found to be the main influence on scores—as one would hope in any valid assessment. However, at least in principle, when criterion-based standard setting is applied, cut-scores should not be directly dependent on the group of candidates sitting an examination, or indeed on other factors such as time of day. The standard is formulated in terms of the hypothetical borderline, or minimally competent, candidate (McKinley and Norcini 2014). Obviously, in practice outcomes of BRM and other examinee-centred approaches do depend on candidate scores (Pell et al. 2010).

### Methods of analysis

We use simple graphical approaches to visualise key variables/relationships (e.g. histograms and error bars). Our main method of analysis is linear mixed effect modelling using the R package lme4 (Bates et al. 2015) (via the function lmer) to estimate the individual effect of each facet in Table 2 on station-level cut-scores.

We begin by analysing individual effects of each facet on cut-scores in three separate simple models (one for each of examiner, station and examination). We then create a combined model for cut-scores including all three of these facets to take account of the fact that each examiner ‘sees’ a potentially unique set of stations, and vice versa.

The formal equation for the combined model is as follows:

$$(Cut - score)_{ijk} = \beta_{0} + examiner_{i} + station_{j} + examination_{K} + \varepsilon_{ijk}$$

where $(Cut - score)_{ijk}$ is the cut-score corresponding to examiner *i*, station *j* and examination *k* (*i *= 1,…547; *j *= 1,…,330; *k *= 1,…,349); $\beta_{0}$ is the grand mean cut-score; $examiner_{i}$, $station_{j}$ and $examination_{K}$ are the random effects of examiner, station and examination respectively on cut-scores (assumed normally distributed); and $\varepsilon_{ijk}$ is the normally distributed error term.

In each of these models, each facet is treated as a random effect. In other words, we are treating the examiners in the sample as representative of the hypothetical universe of all potential examiners. Similarly for stations, and examinations. The model then calculates variance components for each random effect, which tell us how much each facet is contributing to variation in cut-scores across the data.

In the PLAB2 dataset, the station × examiner combination was unique in 5066 (89.7%) of the 6214 administrations, and in only 0.5% of cases was the same examiner present in the same station twice or more. This is important in terms of providing sufficient linking across the data to robustly estimate the different main effects of examiner, station and examination during the estimation process for the combined model. It also means, however, that more complex models, for example, with interaction terms, cannot be robustly estimated.

For each level of the facet (i.e. each examiner, station or examination), the model also produces an estimate of the ‘baseline’ cut-score value for that level. Hence, for examiners this estimate is a measure of the stringency of each examiner in terms of the typical cut-score that they would produce at a typical station in a typical exam. The key benefit of this approach is that the modelling has adjusted for the set stations that the examiner actually ‘sees’ to provide an estimate of cut-score stringency that can be directly compared with that of other examiners.

### Producing ‘fair’ cut-scores at the station and exam level

The model-based station intercept can be thought of as ‘fair’ cut-score for each station—i.e. the cut-score that an examiner of average stringency would produce for that station in a typical exam. In other words, variation in stringency due to a particular examiner in this station in a particular examination has been removed from this fair station cut-score. We can then compare unadjusted cut-scores with fair ones based on the model-based values for each station. This also allows the overall effect of variation in examiner stringency at the examination level to be investigated by aggregating these differences to the examination level.

Note that in producing ‘fair’ scores, we have assumed that variation in cut-score due to *Examiner* and *Examination* facets are error, and that that due to *Station* is not. Methodologically we justify this by considering that we might expect different stations to have different cut-scores since some clinical tasks are inherently more difficult than others. In a conventional psychometric theoretical framework we would not want the same station to have a different cut-score solely because of the examiner assessing it or the examination it is in (Cizek and Bunch 2007, Chapter 2).

To complete comparative analysis of cut-scores, we also analyse differences between unadjusted and fair exam-level cut-scores in terms of the exam-level SEM that is automatically applied to the former to produce the final pass mark for each administration of PLAB2.

## Results

We begin with a graphical representation of station-level cut-score variation by examiner, then present the modelling results, and end with a fair score comparison of examination-level cut-scores.

### Graphical evidence of examiner influences on cut-scores

**Fig. 2:**
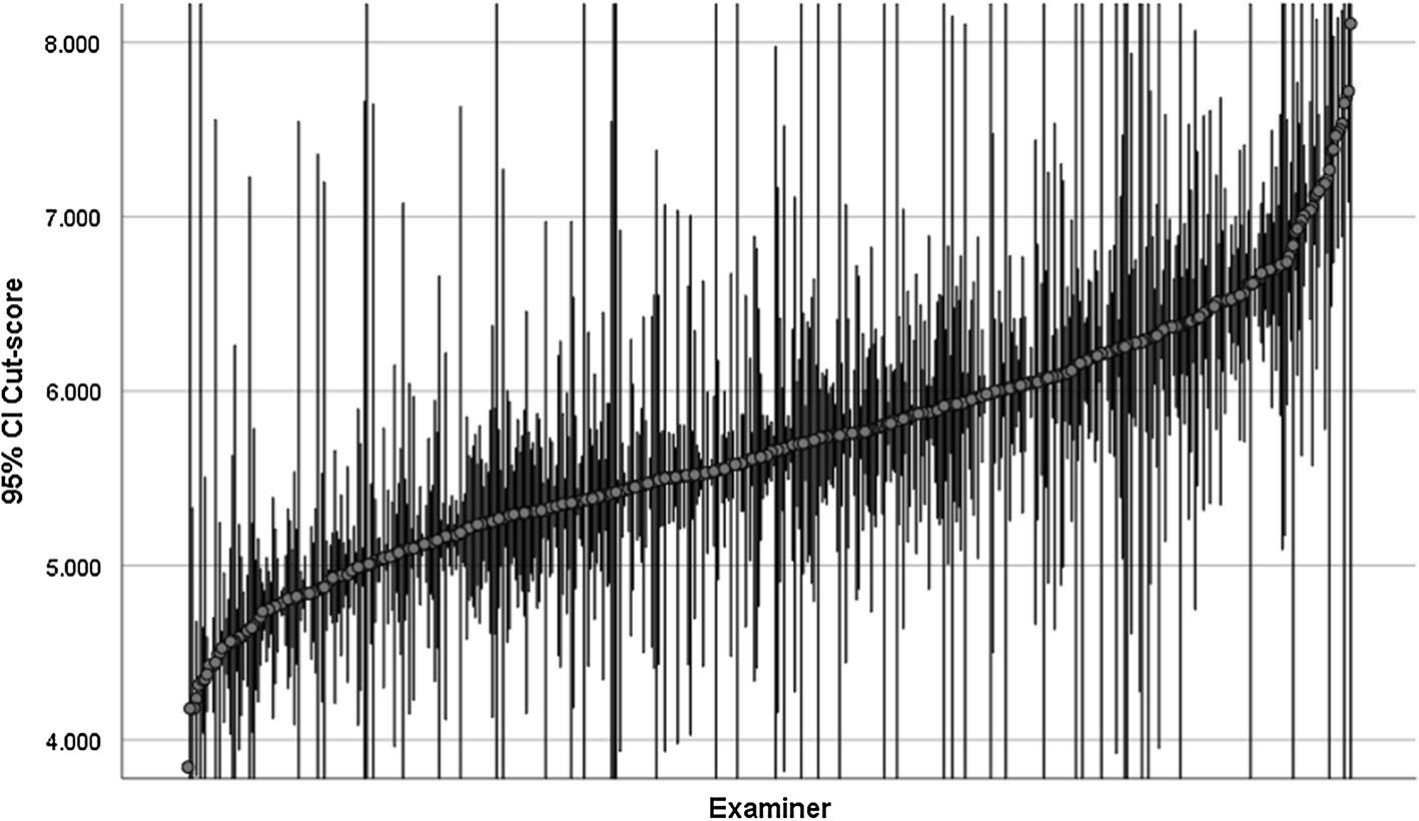
Error bar for mean cut-score by examiner (n = 485)

The variation in cut-scores by examiners is shown in Fig. 2—where each dot is the mean cut-score for the examiner across the data, and the error bars are standard errors for this mean. The error bar is ordered lowest (3.84) to highest mean (8.11).

It is clear from Fig. 2 that there is considerable variation in cut-scores across the set of examiners. Whilst there are some examiners with little data, and hence wide error bars, for most examiners the error bars are relatively small (median standard error = 0.17 marks = 1.4% of the 12 point scale). This simple analysis does not control for the set of stations that the examiner has ‘seen’.

### Three separate models for influences on cut-scores

We first estimate the separate effects of *Examiner*, *Station* and *Examination* on cut-scores. Table 3 gives the results of these three models, and shows that by far the most variation, according to these separate analyses, is due to *Examiner*—57% in this simple model. The other two facets account for much less variation in cut-scores in these single random effect models—8 and 1% respectively for *Station* and *Examination*.

**Table 3:** Three separate random effect model estimates on cut-scores

Model	Facet—single random effect	n	Variance due to facet	Residual variance	Percentage of variance due to single facet
1	Examiner	547	0.371	0.278	57.15
2	Station	330	0.050	0.546	8.36
3	Examination	349	0.007	0.586	1.15

### A combined model for influences on cut-scores

Our key analysis is a combined model that allows for the fact that different examiners ‘see’ different stations, and to control for this in estimating influences on station cut-scores. Table 4 shows that in this model *Examiner* remains by far the most important source of variance in cut-scores.

**Table 4:** Combined random effects model estimates on cut-scores

Facet in combined model	n	Variance due to facet	Percentage of variance due to facet
Examiner	547	0.360	56.13
Station	330	0.003	5.97
Examination	349	0.038	0.41
Residual		0.241	37.49
Total		0.642	100.00

We observe that the estimates are, in broad terms, quite comparable across Tables 3 and 4 but that the precise values have adjusted downward a little in the combined model, as we would expect—any shared variance between facets in the single random effect models is allocated only to one of the facets in the combined model.

In terms of model fit, we see that the residual variance is 37.5%—so the models is doing a good job in explaining the majority of the variance in cut-scores. Further, we note that the combined model residuals are approximately normally distributed (skew = 0.27), and the scatter plot of model predicted and residual values shows no discernible pattern. These are indications that the model is at least adequately representing the data, and has no immediate underlying flaws.

### Unadjusted and fair score comparisons

For each station, the model-based estimate represents a fair value of its cut-score—having stripped out examiner and examination effects. Aggregating these up to the examination level we can compare the relationship between the unadjusted cut-score (i.e. that used in practice) and the model-based fair cut-score. We present the data in this section in percentage terms to account for the fact that not every examination consists of 18 stations.

Table 5 give summary statistics for the two distributions of these cut-scores.

**Table 5:** Summary statistics for examination level percentage cut-scores (n = 349)

	Mean	Standard deviation	Median
Unadjusted exam-level cut-score	46.31	1.67	46.23
Fair exam-level cut-score	47.25	0.26	47.22
Difference = Unadjusted – Fair	− 0.94	1.61	− 1.06

Table 5 shows that the standard deviation in the fair cut-scores is much lower than that in the unadjusted, and this is a natural consequence of variance due to examiners having been removed from the former.

**Fig. 3:**
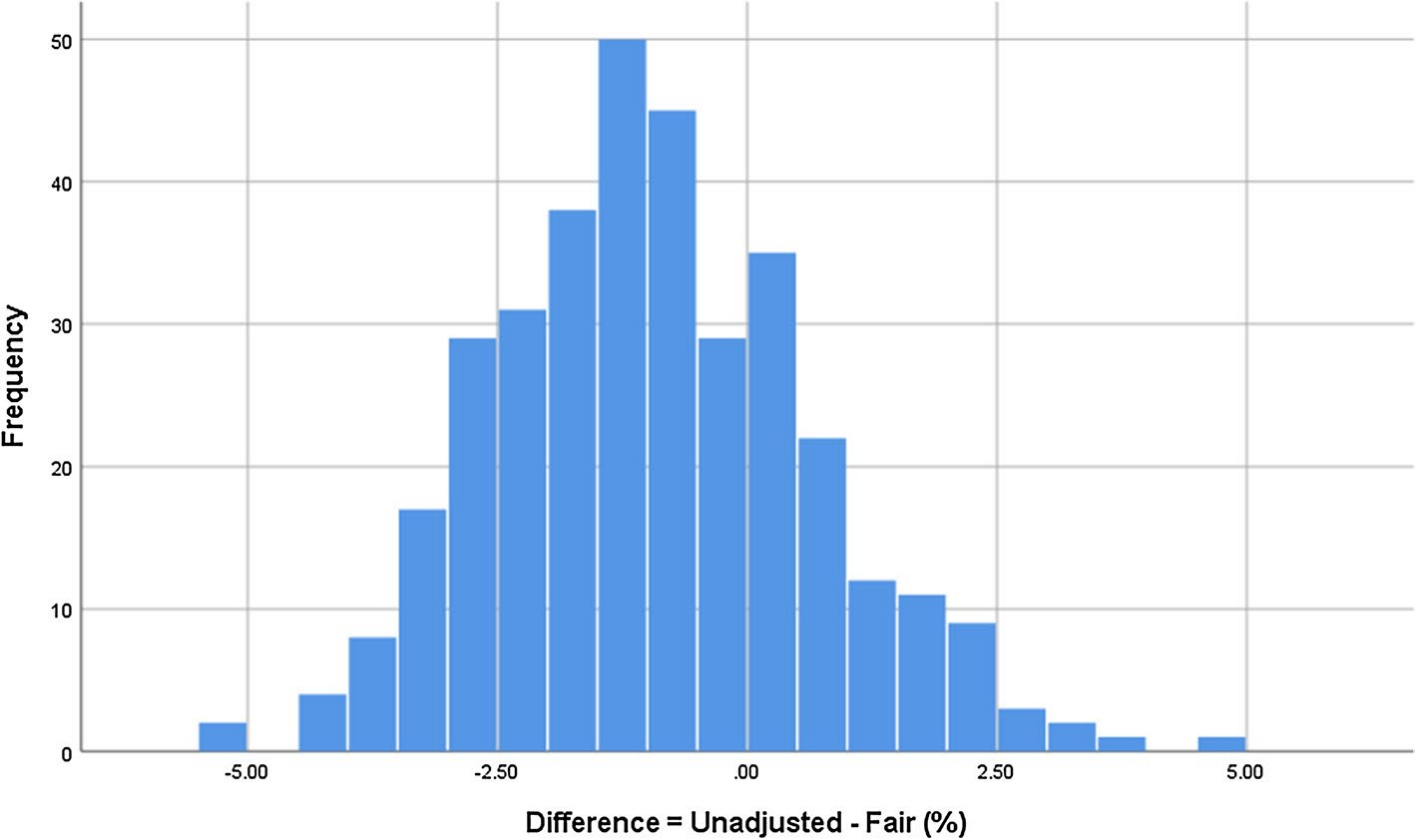
Distribution of differences between unadjusted and fair exam-level cut-scores (%) (n = 349)

Figure 3 shows the full distribution for the differences between these two aggregate level cut-scores.

We can see from Table 5 that fair cut-scores are slightly higher on average than those unadjusted, but Fig. 3 indicates that the impact of examiner stringency is at most 5% in either direction across the 349 PLAB2 administrations. Hence, candidates tend to benefit overall from examiner stringency in their favour—with a slightly lower average value in the unadjusted cut-scores (Table 5).

In PLAB2 the overall passing score is adjusted upwards by a single standard error of measurement (SEM)—which has a mean of 3.6% over the 349 examinations (minimum = 3.0%, maximum 4.5%). When we add the exam-specific SEM to the unadjusted cut-score for that exam to give the final pass mark, we find that only in thirteen exams out of 349 (3.7%) is the fair cut-score higher (i.e. setting a more difficult standard). In other words, the addition of an SEM has ensured that, in the vast majority of PLAB2 exams (96.3%), the final pass mark (unadjusted cut-score plus SEM) provides at least as high a standard as the model-based fair cut-score would.

## Discussion

### The impact of examiners on cut-scores

The main finding of this study is clear—*Examiner is* by far the most important factor in influencing the variance of cut-scores, and much more important than *Station* (accounting for 56% and 6% respectively—Table 4). At face value, the extent of the *Examiner* effect might be seen to undermine the claim that OSCEs are valid and reliable ways to measure clinical performance (Harden et al. 2015, Chapter 3; Khan et al. 2013b). This finding also complements the research on the importance of examiner effects on scoring in OSCEs (McManus et al. 2006; Yeates et al. 2018; Yeates and Sebok-Syer 2017).

We need to carefully consider the extent to which the *Examiner* effect matters in terms of validity of overall assessment outcomes at the exam level. When aggregating up, the analysis suggests that any examiner effect is ameliorated to an extent at the exam level—with ‘error’ impacting only to a degree on the overall passing score (Fig. 3)—similar to findings in other work (Yeates and Sebok-Syer 2017). For those who consider OSCEs as a cornerstone of their assessments (Harden et al. 2015; Khan et al. 2013a, b), this finding might be seen as good news since this partially substantiates, but from a different perspective, the claim that examiner effects to tend to cancel out across at the exam-level, a benefit often claimed of OSCEs (Harden et al. 2015, Chapter 3; Khan et al. 2013b).

In fact, our analysis indicates that ‘error’ in cut-scores in candidates favour—largely as a results of examiner cut-score variance—is typically exceeded by the SEM added to the unadjusted cut-scores to produce the final cut-score in PLAB2. This alleviates the risk of false positive overall decisions in 96% of examinations. The benefit of using the SEM to protect the public from assessment error is clearly demonstrated in this work (Hays et al. 2008; Medical Act 1983).

In terms of alleviating the problem of excessive variation in cut-score stringency, the literature suggests that feedback to examiners on their judgments can sometimes help to reduce (score) stringency (Wong et al. 2020), whilst recognising that this is a complex area and is not always effective (Crossley et al. 2019; Gingerich et al. 2011).The linear mixed modelling automatically produces a measure of cut-score stringency for each examiner, and this could form part of feedback to them of their performance relative to their peers. This information would have to be carefully mediated as it might be difficult for examiners to interpret or act on it compared to the more conventional feedback on scores.

We might not expect *Station* to contribute that much to variance under a criterion-based standard setting regime (Cizek and Bunch 2007, Chapter 1; McKinley and Norcini 2014). In theory at least, the hypothetical borderline student might be expected find all stations broadly of the same level of difficult – although it is known that stations do vary in their difficulty when comparing pass rates and other metrics (Homer et al. 2017). The impact of the *Examination* facet is smaller still (0.4% of variance, Table 4), implying that there is little additional variation in cut-scores across stations within examinations having accounted for variation by *Examiner* and *Station*. Again, this is perhaps what we might expect given the blueprinting and station selection process that goes on in PLAB2 to ensure that test administrations are broadly comparable across a range of factors (General Medical Council 2020b).

### To adjust or not: scores and cut-scores?

The adjustment of candidate scores to fair scores when pass/fail decision-making in an assessment is, ethically, quite difficult to justify. In the literature, this practice is usually modelled but not used for final decision-making (McManus et al. 2006; Yeates et al. 2018). In simple terms, statistical modelling essentially works on the average (Montgomery et al. 2012, Chapter 1), and so whilst we might find that one examiner looks hawkish in their candidate scores according to the modelling, we cannot be sure that the individual scores they have given to a particular candidate on a particular occasion are as a result of this hawkishness. One can certainly argue that overall, decision-making is better (i.e. more accurate) when the score stringency of examiners is adjusted for—that is, after all, the main purpose of carrying out the modelling (Eckes 2011, Chapter 2). However, at the level of each individual, we cannot know if this is the case or not.

Adjusting cut-scores to fair cut-scores, using methods exemplified in this paper, might be seen as less of a fraught issue. This is because any adjustment to cut-scores happens at the station and exam level, rather than at that of the candidate. To implement such an approach needs more consideration, and, this work suggest, would not make a great deal of difference in most examinations—provided the SEM is added when producing the final overall pass mark.

### Study limitations

We briefly consider some limitations of this work.

The issue of the extent to which BRM cut-scores might change when examiner score stringency is adjusted for should be further investigated. This was not possible in this study due to the lack of candidate level data. Such an analysis might require separate estimation of stringency in both in domain scores and global grades—quantifying examiner hawkishness in one, or the other, or both, and assessing how this impacts on standard setting under BRM, and how it might affect measures of examiner cut-score stringency.

Another issue that relates to the lack of candidate scores is that overall PLAB2 pass/fail decisions are also determined by the requirement to pass a minimum number of stations, another under-researched area. Based on the findings of this study, it seems likely that examiner cut-score stringency will impact on pass/fail decisions at the station level, but again will be assuaged to a degree at the exam level. The exact quantification of these effects requires further research.

Whilst this is a ‘high frequency, small cohort’ study analysing a large volume of assessment data, it remains that from a single examination setting in the UK. The evidence base would benefit from attempts at replication of the key study findings (Cai et al. 2018; Makel and Plucker 2014). Given the relatively unique nature of the PLAB2 data, it might prove difficult to find similar types of data from other performance exams that would allow this.

Our modelling, and other literature on examiner stringency (Bartman et al. 2013; Downing 2005; Fuller et al. 2017; Harasym et al. 2008; Jefferies et al. 2007; McManus et al. 2006; Pell et al. 2010; Yeates et al. 2018; Yeates and Sebok-Syer 2017), usually assumes that there is a stable stringency level for each examiner (and each station). The extent to which examiners might develop their practice over time, and how this might impact on cut-scores is unknown, but it is known that untrained examiners, more junior doctors and students tend to mark more leniently (Chong et al. 2017; Khan et al. 2013b) suggesting that experience can change examiner practice—an issue we have not investigated here.

## Conclusion

Using linear mixed modelling approaches, this study has investigated the extent to which cut-scores under BRM vary by examiners (and stations and examinations), and found that at the station level examiner effects are large, but these are greatly weakened, to reach an acceptable level, when aggregated across the exam. In doing so, we have argued for a re-thinking of the concept of examiner stringency—moving away from only considering stringency in examiner scores, towards including the cut-scores that they set in stations via their combined pattern of scoring and grading under BRM.

There remain many complex technical and ethical issues here, particularly when it comes to potentially adjusting scores and cuts-score to make them ‘fair’. These are important areas for future theoretical consideration and empirical research. For now, in totality, the current study, and the principle of parsimony, suggest that when it comes to scoring in OSCEs we might have to accept that professionals will differ in their judgements of performance (including cut-score stringency). We thereby, to a degree, embrace the subjective (Hodges 2013) and acknowledge that a range of factors might influence examiner judgments and that just labelling this as ‘error’ can be simplistic (Gingerich et al. 2011; Govaerts et al. 2007). However, via judicious psychometric approaches (Pearce 2020) including use of the SEM to adjust the exam level pass mark upwards, we evidence in PLAB2 a robust, reliable and generally fair assessment.
